# Supplementary material for: Effects of liraglutide on gut bacterial community dynamics
Source: Microbiol Spectr. 2026 Aug 3;14(9):e00343-26. doi: 10.1128/spectrum.00343-26 (PMC13532357; doi:10.1128/spectrum.00343-26)
Supplement: Supplementary file 1 [file spectrum.00343-26-s0001.docx]

**Supplemental Material**

**Effects of Liraglutide on the Gut Bacterial Community Dynamics**

### Jami Bull^1^, Paul L Durham^1^, Babur S Mirza^1*^

^1^ Department of Biology, Missouri State University, Springfield, Missouri, 65897, USA

*Corresponding author:	Babur S Mirza

Missouri State University

Department of Biology

901 S. National Ave. Springfield, MO 65897

Phone: 417–836–5062

Fax: 417–836–4204

E-mail: baburmirza@missouristate.edu

**Fig. S1**: Boxplots showing the sequence abundance of the nine ASVs in the high-fat control (HFC) group. These nine ASVs were identified as significantly increased in response to 14 days of liraglutide treatment in the HFL group (as shown in Fig. 4), followed by a decline after a one-week washout period. In contrast, the HFC group did not exhibit this pattern; no consistent increase at Day 14 or decrease after the washout period was observed. Each box represents the distribution of sequence counts across eight fecal samples at each time point.

**Fig. S2:** Boxplots showing the mean sequence abundance of the 12 ASVs that were significantly decreased in response to 14 days of liraglutide treatment in the HFL group. These ASVs exhibited a marked reduction at Day 14, followed by a recovery after a one-week washout period (Day 21). Each box represents the distribution of mean sequence counts across eight fecal samples at each time point.

**Fig. S3**: Experimental illustration describing sampling timeline. Overall, 24 male mice, divided into three treatment groups (n = 8 per group): high-fat control (HFC), high-fat liraglutide-treated (HFL), and low-fat control (LFC). Mice in the HFL group received daily liraglutide treatment from Day 0 to Day 14, followed by a one-week washout period (Days 14–21). Fecal samples were collected from all animals in all treatment groups at three time points: Day 0 (baseline), Day 14 (end of treatment), and Day 21 (post-washout). These samples were used to assess liraglutide-induced and diet-related shifts in gut bacterial community structure.

**Table S1**: Represents average body weight, sequencing depth, and alpha diversity metrics across three treatment groups and sampling days. Fecal samples were collected from diet-induced obese mice treated with liraglutide (HFL), high-fat controls (HFC), and low-fat controls (LFC) at baseline (Day 0), after 14 days of treatment (Day 14), and following a 7-day washout period (Day 21). Values represent the mean ± standard error (SE) for each group (n = 8). Alpha diversity was assessed using the Chao1 richness estimator and Shannon diversity index based on 16S rRNA gene sequencing data.

| **Treatment** | **Day** | **Avg. Wt. (g ±SE)** | **Total Seq** | **Avg. Seq/Sample (±SE)** | | | **Chao1 (±SE)** | **Shannon Div. (±SE)** |
| --- | --- | --- | --- | --- | --- | --- | --- | --- |
| HFL (n=8) | 0 | 30.94 ± 0.75 | 679,454 | | 84,931 ± 20,134 | 1,195 ± 157 | | 7.02 ± 0.30 |
|  | 14 | 30.09 ± 0.91^b^ | 668,757 | | 83,594 ± 12,651 | 1,053 ± 161^ab^ | | 5.77 ± 0.26^a^ |
|  | 21 | 33.16 ± 0.10^b^ | 656,203 | | 82,025 ± 8,538 | 1,242 ± 251^a^ | | 6.53 ± 0.50^a^ |
| HFC (n=8) | 0 | 31.20 ± 0.59 | 502,449 | | 62,806 ± 18,038 | 964 ± 203 | | 6.60 ± 0.27 |
|  | 14 | 34.39 ± 0.77^a*^ | 835,795 | | 104,474 ± 21,944 | 1,343 ± 194^a^ | | 6.33 ± 0.53^a^ |
|  | 21 | 36.86 ± 0.97^a*^ | 951,733 | | 118,966 ± 21,834 | 1,325 ± 131^a^ | | 6.51 ± 0.35^a^ |
| LFC (n=8) | 0 | 30.88 ± 0.72 | 758,835 | | 94,854 ± 16,223 | 635 ± 146 | | 5.31 ± 0.45 |
|  | 14 | 31.43 ± 0.58^b^ | 1,074,664 | | 134,333 ± 20,523 | 584 ± 259^b^ | | 4.40 ± 0.50^b^ |
|  | 21 | 32.43 ± 0.63^b^ | 972,369 | | 121,546 ± 24,689 | 462 ± 110^b^ | | 4.55 ± 0.35^b^ |

Values are mean ± SE. Body weight was analyzed by two-way repeated measures ANOVA (group × time) with Tukey’s post-hoc test. Alpha diversity (Chao1, Shannon) was analyzed by Kruskal-Wallis test with pairwise Mann-Whitney U post-hoc (Bonferroni correction). Between-group: superscript letters (^a^, ^b^) indicate significantly different groups within the same day (p < 0.05); groups sharing a letter are not significantly different. Within-group. < 0.05 indicates a significant change in body weight from baseline (Day 0) within the same treatment group (two-way repeated measures ANOVA, Tukey's post-hoc).

**Table S2**: Identification of ASVs that altered in response to liraglutide treatment and showed differential abundance across treatments (HFL, HFC, and LFC groups). We reported closest related organism identified based on 16S rRNA gene identity.

| ASV Number | Closely related organisms based on NCBI blast search | Nucleotide identity (%) | Accession number |
| --- | --- | --- | --- |
| 7 | *Lactobacillus gasseri* strain HL70  *Lactobacillus johnsonii* strain KLDS 1.0731 | 99.76%  99.76% | CP072657  EU626016.1 |
| 8 | *Lactobacillus gasseri* ATCC 33323  *Lactobacillus paragasseri* strain JCM 5343 | 100%  100% | NR_075051 NR_179379 |
| 9 | *Lactobacillus gasseri* ATCC 33323  *Lactobacillus paragasseri* strain JCM 5343 | 99.52%  99.52% | NR_075051 NR_179379 |
| 10 | *Lactobacillus gasseri* ATCC 33323  *Lactobacillus paragasseri* strain JCM 5343 | 99.86%  99.82% | NR_075051 NR_179379 |
| 15 | *Lactobacillus gasseri* ATCC 33323  *Lactobacillus johnsonii* strain KLDS 1.0731 | 99.55%  99.52% | NR_075051 EU626016.1 |
| 1 | *Faecalibaculum rodentium* strain DSM 110083 | 99.76% | NR146011 |
| 3 | *Faecalibaculum rodentium* strain DSM 110083 | 100% | NR146011 |
| 2 | *Faecalibaculum rodentium* strain DSM 110083 | 99.51% | NR146011 |
| 4 | *Faecalibaculum rodentium* strain DSM 110083 | 99.76% | NR146011 |
| 5 | *Faecalibaculum rodentium* strain DSM 110083 | 99.51% | NR146011 |
| 6 | *Faecalibaculum rodentium* strain DSM 110083 | 99.27% | NR146011 |
| 20 | *Duncaniella muris* | 99.31% | NR179975 |
| 21 | *Duncaniella muris* | 99.31% | NR179975 |
| 22 | *Duncaniella muris* | 99.31% | NR179975 |
| 13 | *Dubosiella newyorkensis* strain GB8-5 | 99.51% | OP185270 |
| 14 | *Dubosiella newyorkensis* strain GB8-5 | 99.75% | OP185270 |
| 12 | *Dubosiella newyorkensis* strain GB8-5 | 100% | OP185270 |
| 11 | *Dubosiella newyorkensis* strain GB8-5 | 99.75% | OP185270 |
| 24 | *Acetatifactor muris* | 99.61% | NR117905 |
| 26 | *Acetatifactor muris* | 99.61% | NR117905 |
| 27 | *Acetatifactor muris*  E*isenbergiella* sp. strain C_2 | 100%  99.76 | NR117905  MN081679 |
| 25 | *Acetatifactor muris*  E*isenbergiella* sp. strain C_2 | 99.81%  99.76 | NR117905  MN081679 |
| 36 | *Leptogranulimonas caecicola* strain TOC12  *Olsenella phocaeensis* strain Marseille-P2936 | 99.85%  99.75% | NR_184626  NR_173693 |
| 37 | *Leptogranulimonas caecicola* strain TOC12  *Olsenella phocaeensis* strain Marseille-P2936  Granulimonas faecalis | 99.81%  99.51%  99.32% | NR_184626  NR_173693  NR_184625 |
| 32 | *Romboutsia ilealis* strain CRIB | 99.51% | NR140740 |
| 41 | *Leptogranulimonas caecicola* strain TOC12 *Olsenella phocaeensis strain* Marseille-P2936  *Leptogranulimonas caecicola* strain TOC12 | 100%  100%  100% | NR_184626.1 NR_173693  MK929055 |
| 33 | *Romboutsia ilealis* strain CRIB | 99.76% | NR140740 |
| 34 | *Romboutsia ilealis* strain CRIB | 99.76% | NR140740 |
| 39 | A*topobiaceae bacterium* strain 61-8  *Leptogranulimonas caecicola* strain TOC12  *Muricaecibacterium torontonense* strain NM07_P-09 Olsenella sp. Mou02 | 99.75%  99.75%  99.75%  99.75% | PP512922  NR_184626  MK929055  KM405314 |
| 45 | *Lactococcus lactis* strain CAU:1432 | 99.51% | MF354960 |
| 46 | *Faecalicatena fissicatena* | 99.47% | LC483565 |
| 51 | *Faecalicatena fissicatena* | 96.84% | LC483565 |
| 52 | *Clostridiales bacterium* CIEAF 017 | 99.51% | AB702931 |
| 56 | *Clostridiales bacterium* CIEAF 017 | 100% | AB702931 |
| 57 | *Clostridiales bacterium* CIEAF 017 | 100% | AB702931 |
| 59 | *Clostridiales bacterium* CIEAF 017 | 99.76% | AB702931 |
| 82 | *Oscillibacter* sp. strain 128x | 99.27% | MK287653 |
| 85 | *Oscillibacter* sp. strain 128x | 99.51% | MK287653 |
| 44 | *Lactococcus lactis* strain CAU:1432 | 99.76% | MF354960 |
